# Favourable outcome of *Fusarium* prosthetic valve endocarditis in a patient with an Ebstein anomaly

**DOI:** 10.4102/sajid.v40i1.699

**Published:** 2025-02-11

**Authors:** Scott G. Lee-Jones, Stefan van der Westhuizen, Johannes Taljaard, Nelesh P. Govender, Rubina Razack

**Affiliations:** 1 Division of Infectious Diseases, Department of Medicine, Faculty of Medicine and Health Sciences, Stellenbosch University, Cape Town, South Africa; 2 Division of Cardiothoracic Surgery, Faculty of Medicine and Health Sciences, Stellenbosch University, Cape Town, South Africa; 3 School of Pathology, Faculty of Health Sciences, University of the Witwatersrand, Johannesburg, South Africa; 4 Medical Research Council Centre for Medical Mycology, University of Exeter, Exeter, United Kingdom; 5 Division of Anatomical Pathology, Faculty of Medicine and Health Sciences, Stellenbosch University, Cape Town, South Africa

**Keywords:** endocarditis, invasive fungi, *Fusarium*, fusariosis, Ebstein anomaly

## Abstract

Fungal endocarditis is rare, challenging to diagnose and treat and associated with high mortality. We describe a case of *Fusarium solani* endocarditis in a patient with Ebstein’s anomaly with a favourable outcome.

**Contribution:**

We emphasise that surgical intervention with source control of the infective vegetation is still the mainstay of treatment and highlight the importance of submitting infected source material for histology, culture and molecular testing to identify the causative organism.

## Introduction

Fungal pathogens are uncommon but emerging causes of infective endocarditis, accounting for 1% – 2% of all cases and up to 4% of those involving a prosthetic valve.^1,2,3^ The low incidence, non-specific clinical symptoms, difficulty in identifying the fungus, as well as challenges with antifungal therapy contribute to the high mortality rate associated with fungal endocarditis.^1,2,3,4^ A spectrum of moulds, yeasts and dimorphic fungi can cause fungal endocarditis. *Candida* spp. account for up to two-thirds of all cases, followed by *Aspergillus* spp. and *Histoplasma capsulatum*.^1,3^ Rarer fungal pathogens, such as *Fusarium* spp. comprise the remainder.^1^ *Fusarium* spp. are infrequently reported as aetiological agents of endocarditis, but their global incidence is increasing.^4^

We describe a case of *Fusarium solani* endocarditis developing in a prosthetic tricuspid valve inserted for an Ebstein’s anomaly. We aim to raise awareness of this life-threatening fungal infection and present explanations for the course of events in our patient’s care, which led to a good clinical outcome.

## Case presentation

A 26-year-old woman required insertion of a bioprosthetic tricuspid valve on 04 May 2023 for severe symptomatic tricuspid regurgitation secondary to an underlying congenital Ebstein’s anomaly first diagnosed at two years of age. She had no other relevant comorbid illnesses and was not an intravenous drug user. Six months post-surgery, she developed fever, a productive cough and shortness of breath requiring consecutive hospital admissions in November and December. She responded poorly to standard empiric antibiotic therapy for community and hospital-acquired pneumonia with ceftriaxone 1g daily intravenously (IV) for 5 days and piperacillin-tazobactam 4.5 g three times daily IV together with Amikacin 750 mg daily for 5 days, respectively. Investigations for a progressive non-resolving pneumonia were conducted. Blood cultures collected on both admissions were negative. No organisms were observed on microscopy from a bronchoalveolar lavage (BAL) and only *Candida* spp. (considered a commensal organism of the upper respiratory tract) was cultured. Tuberculosis nucleic acid testing of BAL fluid (Xpert MTB/RIF Ultra, Cepheid) was negative for *Mycobacterium tuberculosis* complex. A chest computed tomography (CT) scan showed lobulated peripheral peri-bronchial air space opacification with central ground glass attenuation predominantly in both lower lobes, suggestive of a bilateral lower lobe organising pneumonia. Corticosteroid therapy was commenced (prednisone 1 mg/kg) on 22 December 2023, after which she was discharged for outpatient follow-up.

Although some improvement in symptoms was reported, symptoms recurred around mid-January 2024 together with a papular rash involving the face, trunk and both arms. Her condition rapidly deteriorated with marked fever and respiratory symptoms necessitating readmission for high care and specialist input.

**FIGURE 1:**
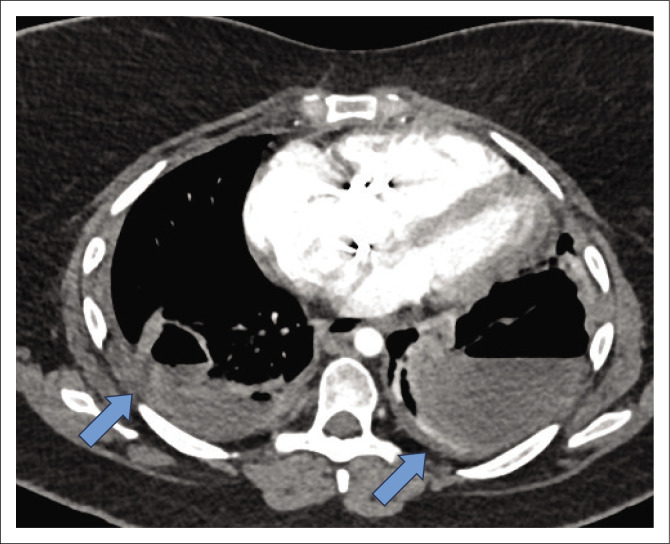
Axial view of computed tomography chest demonstrating bilateral lung abscesses, most notably on the left.

**FIGURE 2:**
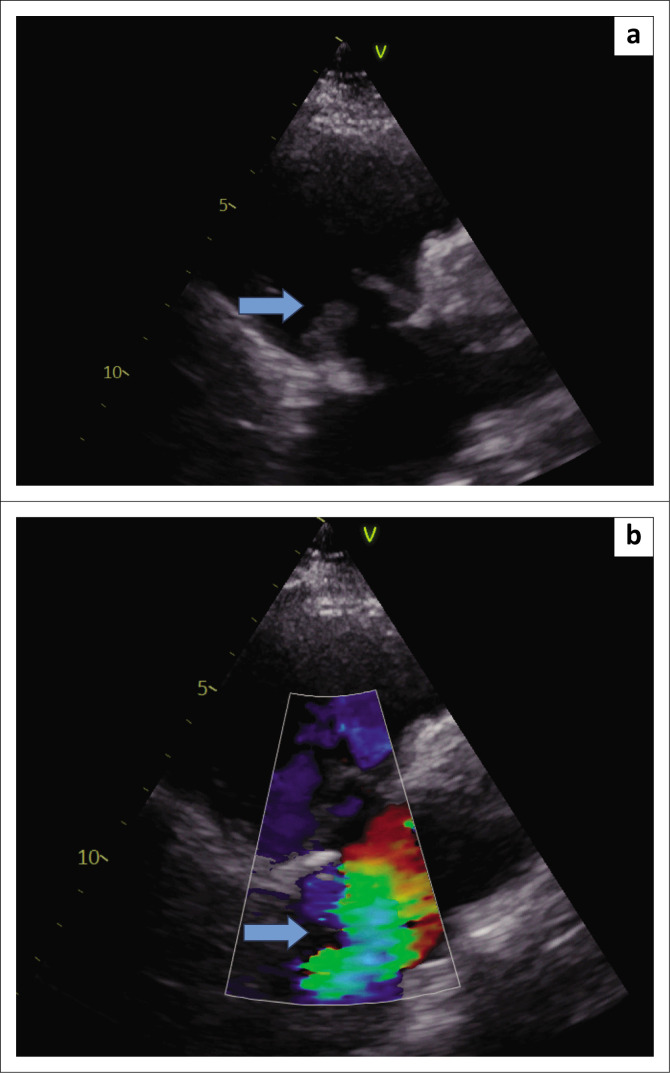
(a) Right ventricular outflow tract view demonstrating large vegetations on the tricuspid valve prosthesis, and (b) colour of same view showing severe tricuspid regurgitation.

Her general examination was normal, and the reported skin rash was not clearly visible. Bilateral coarse crackles were audible with amphoric breathing in the left mid-zone. Notably, no cardiac murmur or features of heart failure were present. Bilateral lung abscesses were found on chest radiograph. Broad antimicrobial cover with intravenous (IV) ertapenem 1g daily IV and vancomycin 1.5g IV was commenced for a nosocomial infection, but vancomycin was discontinued after a single loading dose. She responded well to antimicrobial therapy and a repeat chest CT (Figure 1) confirmed bilateral lung abscesses and loculated empyemas. Large vegetations were noticed on her tricuspid bioprosthesis on transthoracic echocardiography (TTE) (Figure 2). A single blood culture set from this admission was negative.

The infective endocarditis team elected to change her antibiotic therapy to vancomycin guided by therapeutic drug monitoring and ceftriaxone 2 g daily plus rifampicin 600 mg twice daily to treat her right-sided prosthetic valve infective endocarditis complicated by bilateral embolic lung abscesses. She received antibiotics for 21 days (including initial nosocomial and targeted empiric) prior to urgent cardiac surgery during which the infected tricuspid prosthesis and large adherent vegetations were removed. A 29 mm Edwards Perimount valve was implanted into the annulus.

**FIGURE 3:**
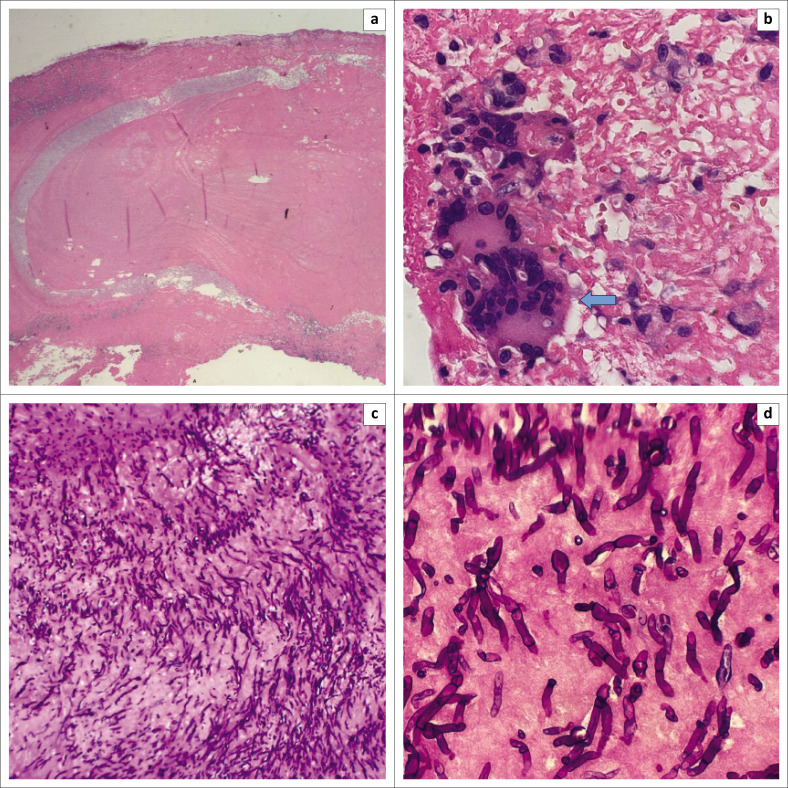
(a) nodular fragment with cleared halo at the rim, (b) Multinucleated giant cells, (c) APAS stain highlighting numerous fungal hyphae, and (d) Septate hyphae.

The infected valve tissue was submitted for culture, 16s & 18s ribosomal ribonucleic acid (rRNA) polymerase chain reaction (PCR) assays and histopathological evaluation. Haematoxylin and eosin (H&E) stained sections revealed necrotising granulomatous inflammation and abundant branching septate hyphae. The hyphae were highlighted with Periodic acid-Schiff (PAS) staining. These features are all demonstrated in Figure 3. No native or prosthetic valvular tissue was identifiable microscopically.

*Fusarium* sp. was cultured from the excised valve tissue and subsequently speciated as *F. solani* on 18s PCR sequencing. Post-operatively there was a dramatic clinical improvement. The patient self-reported rapid clearing of the papular lesions post-surgery.

The abscesses had largely resolved on antibiotics and postural drainage, and the corticosteroid therapy was discontinued after a new diagnosis of diabetes mellitus was made (HbA1c of 8.1%) and together with the provisional microscopy features suggesting fungal endocarditis.

Intravenous therapy with amphotericin B deoxycholate (AmB-d) at 1 mg/kg daily and voriconazole (VCZ), (6 mg/kg twice daily for 24 h then 4 mg/kg twice daily) was commenced after the PCR result became available and antibiotic therapy was discontinued. The decision to discontinue antibiotic therapy was supported by the absence of any previous positive blood cultures, the negative bacterial PCR test and the concern for renal toxicity with multiple nephrotoxic antimicrobial agents.

As mild acute kidney injury occurred after 4 doses of AmB-d (peak creatinine 222 µmol/L), this was discontinued whereafter renal function returned to normal baseline. It was decided to continue treatment with VCZ alone for a total duration of 12 weeks. The patient remained well and was switched to oral VCZ after 2 weeks and discharged home. At her 1-month follow-up visit, she was asymptomatic. Transoesophageal echocardiogram (TOE) performed 1 week prior to completion of VCZ was normal, with no vegetations or other notable abnormality.

## Discussion

Ebstein’s anomaly is a rare congenital malformation of the tricuspid valve with myopathy of the right ventricle, necessitating surgery at some point in the patient’s life.^5,6,7^ Long-term survival after surgery is favourable, but adverse effects can occur, including prosthetic valve infective endocarditis as seen in this case. To date, only five cases of Ebstein’s anomaly-associated endocarditis have been reported: all with bacterial causes.^5,6,8,9,10^ We describe the first case of confirmed *Fusarium* endocarditis in the setting of an Ebstein’s anomaly. Survival rates of *Fusarium* endocarditis are poor, the mortality rate reaching 80%.^11,12,13,14,15,16,17,18,19,20^ To our knowledge, only two cases have documented survival in published reports thus far.^2,21^ Most of these cases had underlying conditions causing immunosuppression, either because of haemato-lymphoproliferative disorders or immune suppressive therapy for organ transplants and other reasons, while few developed *Fusarium* endocarditis after coronary artery bypass grafting, and one on a prosthetic aortic valve.^11,12,14^ Transient immunosuppression from high-dose oral corticosteroids, newly diagnosed uncontrolled diabetes mellitus, broad spectrum antibiotics and a prosthetic valve are all contributing risk factors for invasive fungal disease in our patient.^1^ The development of early fungal prosthetic valve endocarditis in this case may also have resulted from colonisation of the bioprosthesis prior to implantation. Most bioprosthetic valves are disinfected with glutaraldehyde, which may not be entirely effective against *Fusarium* spp.^22^

*Fusarium* spp. are hyaline filamentous fungi found ubiquitously in nature. They can cause a broad spectrum of infections in humans, termed fusariosis.^23,24^ The clinical form depends on the portal of entry and the immune status of the host. Immunocompromised patients are more likely to present with disseminated disease and immunocompetent patients usually have localised disease limited to the skin and eyes, but can also occasionally develop sinusitis, pneumonia and fungaemia.^23,24^ A common manifestation in immunocompromised patients is multiple disseminated painful erythematous papular or nodular skin lesions.^23^ The resolving skin lesions reported by our patient may have been related to fusariosis although this was not confirmed by culture or histological examination of skin tissue. Innate immunity plays a major role in resisting such mould infections.^23,24^ *Fusarium* is relatively virulent, producing mycotoxins which can further suppress humoral and cellular immunity, cause tissue breakdown and angioinvasion. They also have an affinity to adhere to prosthetic material.^24^

This case demonstrates challenges with diagnosis of fungal infections. A transbronchial biopsy at the time of the BAL could have identified the cause of the organising pneumonia. *Candida* spp. are frequent colonisers grown on respiratory specimens and rarely cause of pulmonary infection.^25^ We postulate that fungal emboli to the lungs acted as a nidus, predisposing the patient to polymicrobial superinfection, causing her lung abscesses. This could explain the improvement in her lung abscesses on antibiotic therapy.

The immunosuppressive effect of corticosteroids in this case was profound leading to unchecked fungal overgrowth. The histology, which showed poorly formed granulomas with decreased numbers of macrophages in the vegetation, supports these findings. Corticosteroid administration during invasive fungal disease is associated with an increased mortality, and therefore avoiding or minimising it improves the outcome of most life-threatening fungal diseases.^26^ Immediate cessation of the steroids and early removal of the vegetation (and therefore the immune-inhibiting mycotoxins) most likely restored the patient’s cellular and humoral immunity contributing to the favourable clinical outcome.

Fungal organisms are recognised causes of culture negative endocarditis. In a prospective cohort of infective endocarditis cases in the Western Cape, South Africa using a set protocol, only 2% of cases were fungal.^27^ Local practice therefore does not include antifungal treatment in culture negative endocarditis unless other compelling indications are present. Sending the explanted valve for histopathological evaluation, culture and molecular testing are important modalities in making a final diagnosis of fungal endocarditis. This is especially so if features of granulomatous inflammation are present on histology.

Ideal treatment of this case was challenging. *Fusarium solani* is intrinsically resistant to echinocandins and most azoles.^28^ In the absence of any randomised trial data to guide the treatment of invasive fusariosis, monotherapy with either VCZ, AmB-D, liposomal amphotericin B or combination therapy with VCZ plus amphotericin B is recommended.^29^ Both cases of *Fusarium* endocarditis with documented survival were treated with combination therapy using amphotericin B together with terbinafine or VCZ, respectively.^2,21^ As a result of limited access to liposomal amphotericin B, we elected to complete treatment with VCZ monotherapy. As no established minimum inhibitory concentration breakpoints exist for VCZ to determine susceptibility, combination therapy remains the guideline recommendation.^29^

## Conclusion

This rare case of *F. solani* endocarditis in a woman with a bioprosthetic tricuspid valve for an underlying Ebstein’s anomaly highlights several key learning points: (1) invasive sampling (e.g. BAL with lung biopsy) may have a role in earlier diagnosis, (2) the importance of source control and/or surgical debridement where applicable, (3) submission of the infected source material for histology, culture and molecular testing to increase the likelihood of definitive diagnosis and (4) the improved outcome with minimising or avoiding corticosteroids.
